# Report of the Diseases of Edinburgh, for August 1808

**Published:** 1808-10

**Authors:** John Roberton

**Affiliations:** Surgeon


### *Report of the Diseases of Edinburgh, for August* 1808; *by* Mr. John Roberton, *Surgeon.*

IN this part of the world we are usually visited in the beginning of August by heavy falls of rain, known to the inhabitants under the name of the Lammas Flood. These, in general, continue only a few days; but when, as in the present season, they continue longer, which they did for the first fortnight of the month, they are generally productive ductive of much injury to the crops. Besides, the exhalations from the ground during the foggy days; and the damp, chill, and disagreeable state of the air after sunset, at this time, render the existence of the infirm extremely irksome. Those too, who are subject to the periodical recurrence of slight chronic complaints, have, during such unfavourable states of the weather, each attack considerably aggravated. The weather, however, about the middle of the month, became pleasant, and, for the most part, continued so till the end of it. Although the grain also had seemingly suffered much from rain, the winds, that almost immediately followed, did them great service; and the harvest, by the end of the month, was in considerable forwardness. The thermometer has, especially in the day, indicated from 60 to 70, and sometimes even 80 degrees of heat; and the barometer, even during the heavy rains, never fell so low as often observed when the air is apparently more humid. Some gales of wind, toward the end of the month, seemed to have greater effect in lowering the barometer than even the rains.

Diarrhœa still continues with considerable violence; indeed, with the addition of cholera, it constitutes the principal disease for August. Last month, diarrhœa continued to increase in severity; and about the end of it, or early in the present month, this disease, in some instances, gradually and almost imperceptibly assumed the symptoms of cholera. Thus, probably, from a peculiarity of constitution of the individuals, some were seized with the one, and some with the other of these complaints; while others were first seized with diarrhœa, and afterward with cholera. Although these complaints have been obstinate, I have not met with any that have proved fatal. One or other of them, however, having in some of my patients supervened during a hectic state of the system, have undoubtedly terminated the existence of some of the consumptive, which otherwise might have been prolonged for a few weeks or months.

Diarrhœa, in the present state of it, seems for the most part to owe its origin to substances lodged in the stomach and intestines, and thus probably predisposing to cholera, particularly if free evacuation be not attended to early in the disease. When cholera has formed, opiates and emollient glysters seem best suited to its removal. In some cases of diarrhœa, I have found the administration of different kinds of purgative medicines quite ineffectual, particularly when the disease, as is often the case at present, happens happens to be combined with ascarides. I have, in such instances, given submuriate of mercury and assafœtida, in the form of pills, with the very best effect. Sometime ago a case of this kind, and rather of a curious nature, came under my care. The patient was a man about thirty years of age, by trade a smith, who had for two years previously been frequently afflicted with violent diarrhœa, for the removal of which he, by the advice of a physician in Edinburgh, used at one time astringent mixtures, at another smart cathartics, composed of submuriat. hydrarg. and jallap; but neither of these produced any permanently beneficial effects. Receiving, however, momentary relief from them, he occasionally persevered in their use: he, nevertheless, became pale, emaciated, and unable to work at his trade; and in this state I first saw him. He complained of pain about the region of the stomach and bowels, and itching about the anus, with diarrhœa and constipation alternating. Upon taking a dose of the above purgative medicine, he, as formerly, evacuated small parcels of ascarides; and afterwards, for a day or two, even without medicine, he voided several of them; but still derived no lasting relief. I then prescribed for him, pills composed of three grains of assafœtida and two of submuriat. hydrargyrus, desiring him to take two of them thrice a day. It was not till after the third day that he had any considerable evacuation in consequence of them, when, to prevent the probable effects of such large quantities of calomel on the general system, I desired him to desist, and ordered for him half a drachm of pulv. jallap. During the following day, the immense quantities of dead ascarides, which he at several times evacuated, were incredible. Indeed, I could not, previously, have conceived it possible for such numbers to remain in the intestinal canal, without its sustaining almost irreparable injury.

Notwithstanding the debility of this patient, I followed up the first doses of the calomel and assafœtida with still larger doses of the same medicine, and principally supported him by nourishing soups, and such cordials as his stomach seemed capable of receiving, without rendering him sick. Still fresh loads of ascarides were discharged; and in this way he continued the medicine nearly six weeks, when no more of the ascarides were evacuated, and I ceased to administer any medicine. I lastly ordered for this patient, wine, porter, soups, &c. in such quantities as not to oppress his digestive organs; and he is now a stout man, man, completely free from every complaint, and works at his trade with the utmost ease.

From the result of the above case, I have uniformly, on the present occasion, administered the same medicine with invariable success, and even to young children.

Intermittent fever, although, according to my former statements, of late a very uncommon disease in this place, has recently come under my observation in several instances. I have met with it both among adults and children, and have uniformly removed it, except in one instance, by administering the Peruvian bark. I was induced by the obstinacy of one of the cases alluded to, to remove the patient a few miles into the country, when, even without the bark, he recovered completely in a fortnight. The ground where he was first affected was not marshy, but there was a collection of ordure at a short distance from his house, the effluvia of which, in all probability, occasioned his disease.

I think it my duty to mention in this place, that, though not epidemic diseases, I have lately met with an immense number of cases of very great debility of the generative organs, with, and sometimes without, seminal emissions.— Till lately, I never imagined that this constituted a disease of such a formidable nature, as those cases which have come under my observation have proved to me. The penis shrunk, one or both testicles diminished in size, the spermatic chord and epididymus enlarged, partial blindness, cold extremities, with continual horror of mind, convey but a faint idea of the state into which I have seen many patients reduced. In such a dreadful state, I have, however, by the properly regulated use of cantharides, completely restored such patients to health. As I consider these cases of the greatest importance, I shall, in the intervals of professional engagements, draw up a plain statement of them, and through the medium of the Journal, or otherwise, submit them to the public.

*No. 4, St. James's Street.*

